# Comparison of Postnatal Growth Charts of Singleton Preterm and Term Infants Using World Health Organization Standards at 40–160 Weeks Postmenstrual Age: A Chinese Single-Center Retrospective Cohort Study

**DOI:** 10.3389/fped.2021.595882

**Published:** 2021-03-15

**Authors:** Li Zhang, Jian-Gong Lin, Shuang Liang, Jin Sun, Nan-Nan Gao, Qiong Wu, Hui-Yun Zhang, Hui-Juan Liu, Xiang-Deng Cheng, Yuan Cao, Yan Li

**Affiliations:** ^1^Department of Developmental Pediatrics and Child Health Care, The First Affiliated Hospital of Shandong First Medical University, Shandong Provincial Qianfoshan Hospital, Shandong Engineering and Technology Research Center for Pediatric Drug Development, Jinan, China; ^2^Department of Nephrology, Shandong Provincial Hospital Affiliated to Shandong First Medical University, Jinan, China; ^3^Department of Pediatrics, The Second Hospital of Shandong University, Jinan, China; ^4^Department of Pediatrics, Maternal and Child Health Care Hospital of Shandong Province, Jinan, China

**Keywords:** preterm infants, term infants, singleton, postnatal growth, growth assessment, growth charts, WHO standards

## Abstract

There remains controversy regarding whether the growth charts constructed from data of term infants, such as those produced by the World Health Organization (WHO) standards, can appropriately evaluate the postnatal growth of preterm infants. This retrospective cohort study, conducted in the First Affiliated Hospital of Shandong First Medical University in Jinan China, aimed to compare the postnatal growth charts of singleton preterm and term infants using WHO standards at 40–160 weeks postmenstrual age (PMA). A total of 5,459 and 15,185 sets of longitudinal measurements [length/height, weight, head circumference (HC), and body mass index (BMI)] from birth to 160 weeks PMA were used to construct growth charts for 559 singleton preterm (mean PMA at birth, 33.84 weeks) and 1,596 singleton term infants (born at 40 weeks PMA), respectively, using the Generalized Additive Models for Location, Scale, and Shape (GAMLSS) method. Z-scores (prematurity corrected) were calculated using WHO Anthro software. Compared to WHO standards, all parameters of preterm infants were increased, especially in terms of length/height and weight; the gap between the two almost spanned two adjacent centile curves. Compared to term controls, the length/height, weight, and BMI of preterm infants were higher at 40 weeks PMA, surpassed by term infants at 52–64 weeks PMA, and quite consistent thereafter. The HC of preterm infants at 40–160 weeks PMA was quite consistent with both term controls and the WHO standards. The Z-scores for length/height, weight, and BMI of preterm infants relative to the WHO standards gradually decreased from 1.20, 1.13, and 0.74 at 40–44 weeks PMA to 0.67, 0.42, and 0.03 at 132–160 weeks PMA, respectively; Z-scores for HC of preterm infants rapidly decreased from 0.73 to 0.29 at 40–50 weeks PMA, and then fluctuated in the range of 0.08–0.23 at 50–160 weeks PMA. Preterm infants had higher growth trajectories than the WHO standards and similar but not identical trajectories to term infants during the first 2 years of life. These findings reemphasize the necessity of constructing local growth charts for Chinese singleton preterm infants.

## Introduction

Growth impairment during early postnatal life can have permanent detrimental effects in later life, such as short stature, high blood pressure, and impaired neurodevelopment (1–4). A full understanding of optimal postnatal growth is of critical importance for improving survival and long-term outcomes in preterm infants (2). This requires robust growth charts to monitor whether preterm infants have potentially abnormal growth that might be indicative of adverse health conditions (5). Recently, there has been an increase in the number of internationally validated growth charts for tracking infant growth, including charts for term infants and preterm infants (6, 7). However, the selection of growth charts has always been controversial given the lack of consensus regarding the most suitable charts to use (8). The most prominent of these growth charts are the World Health Organization (WHO) growth standards (9), which are widely used to evaluate the postnatal growth of preterm infants after they reach corrected term age due to the scarcity of high-quality growth charts for preterm infants. However, there has been concern that the WHO standards may not be suited for evaluating the postnatal growth of preterm infants and that, in using them, we may misdiagnose either poor or excessive growth (10).

To clarify the postnatal growth of singleton preterm infants and whether the WHO growth charts can adequately and appropriately evaluate the postnatal growth of preterm infants in Jinan, Shandong Province, China, during the first 2 years of life, we constructed postnatal growth charts using longitudinal data from a specific cohort of singleton preterm infants during the first 2 years of life. These data were compared to data from a cohort of singleton term infants in the same center and the WHO standards (9).

## Materials and Methods

### Study Design

Longitudinal growth data were retrospectively collected from an ongoing cohort study for preterm infants and from routine well-child visits for term infants conducted in the Department of Developmental Pediatrics and Child Health Care, the First Affiliated Hospital of Shandong First Medical University (a tertiary public hospital) in Jinan, Shandong Province, China.

### Inclusion and Exclusion Criteria

**Figure 1:**
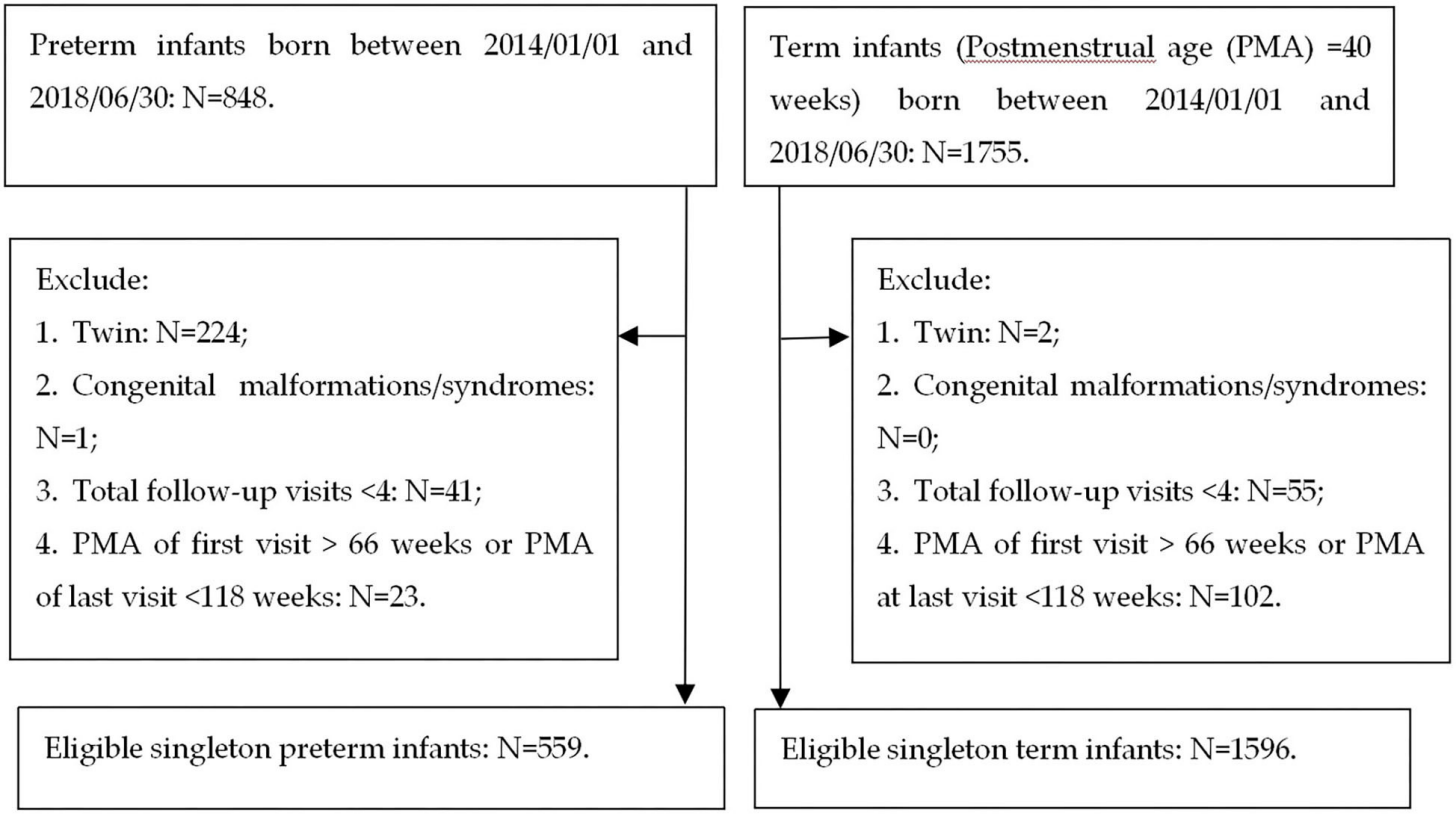
Flow charts of recruitment of singleton preterm and term infants.

The inclusion criteria of subjects were as follows: (1) preterm infants [postmenstrual age (PMA) at birth ≤ 36 weeks] and term infants (PMA at birth = 40 weeks) born between January 1, 2014, and June 30, 2018; (2) singleton; (3) no congenital malformations or syndromes; (4) ≥4 follow-up visits before 160 weeks PMA; and (5) PMA at first follow-up visit ≤ 66 weeks (equivalent to a corrected age of 6 months) and PMA at last visit ≥118 weeks (equivalent to a corrected age of 18 months). The exclusion criteria were as follows: (1) twins; (2) infants with congenital malformations or syndromes; (3) infants with a first follow-up visit after 66 weeks or lost to follow-up before 118 weeks PMA. A flowchart of the sampling process for eligible singleton preterm and term infants is shown in Figure 1. Ethical approval was obtained from the Medical Ethics Committee of the First Affiliated Hospital of Shandong First Medical University. Informed consent was obtained from the parents of each infant.

### Feeding Practices

The feeding practices during the early postnatal period for preterm infants were based on the “Nutrition Practice Care Guidelines for Preterm Infants in the Community 2013” and “Chinese Society of Parenteral and Enteral Nutrition (CSPEN) guidelines for nutrition support in neonates” (11, 12). Briefly, before discharge, the preterm infants were fed according to their nutrition risks, as described in detail in our previous study (13). After discharge (when infants weighed ≥2,000 g target weight, with stable feeding and body temperature), the parents were encouraged to feed their babies with breastmilk without fortification (standard infant formula was used in cases of insufficient breastmilk). Introduction of supplementary food was recommended from a corrected age of 4–6 months. It was recommended that the term infants should be exclusively breastfed before 4–6 months, at which point supplementary food could be introduced. The adherence to the above recommendations was documented in questionnaires at each follow-up visit. If there was undesirable adherence, we would further ask the causation and gave individual recommendations to verify adherence to feeding practices.

### Baseline Data Collection and Anthropometric Measurements

The parental baseline data collected were as follows: age, education, ethnicity, maternal obstetric history, and mode of delivery. The data on the infants were as follows: sex, PMA at birth (week), and length (cm) and weight (kg) at birth [head circumference (HC) was not measured at birth]. These data were collected from questionnaires filled in by parents at the first follow-up visit. Except for parental education, other information obtained through questionnaires was further verified by birth certificates or medical records. If there were discrepancies, data from birth certificates or medical records were chosen to be documented.

It was recommended that infants should be followed up at term (40 weeks PMA) and at 1, 3, 4, 5, 6, 9, 12, 15, 18, and 24 months of age (corrected age for preterm infants). Anthropometric measurements were taken at each follow-up visit by two trained staff members working in pairs. For children ≤ 2 years old (corrected age for preterm infants), the length was measured with the infant lying down and using an infantometer (“KANGWA” WS-RTG-1G, Suzhou, China; range 30–105 cm, with digit counter readings precise to 1 mm). For children > 2 years old (corrected age for preterm infants), and height was measured with the infant standing upright, using a stadiometer (“BeiSiTe” SZG-180/210, Changzhou, China; range 30–180 cm, with digit counter readings precise to 1 mm). Weight was measured using an electronic scale (“KANGWA” WS-RTG-1G, Suzhou, China; range 0–60 kg; calibrated to 0.05 kg). HC was measured with a tape measure (“WenTai,” Infant HC Tape Measure, Foshan, China; range 0–56 cm, with digit counter readings precise to 1 mm), which was replaced once a month. Each staff member independently measured and recorded a complete set of measurements. Thereafter, the two staff members compared their readings and recorded the mean of each pair of readings. The maximum allowable differences were as follows: length/height, 7 mm; weight, 100 g; and HC, 5 mm. If there were greater discrepancies, both staff members independently measured and recorded a second and, if necessary, a third set of readings for the variable (s) in question.

The Z-scores of length/height, weight, HC, and BMI (demonstrated as HAZ, WAZ, HCZ, and BAZ, respectively) of both the preterm and term infants obtained from each follow-up visit were calculated using WHO Anthro (9) in R software (version 3.6.1). Preterm infants were corrected for prematurity in the calculation of the Z-scores.

The Z-scores of length and weight at birth according to the INTERGROWTH-21st International Newborn Size References/Standards (INSR/S; 24–42 weeks PMA) were calculated using the International Newborn Size at Birth Standards application (14).

### Construction of Growth Charts for the Preterm and Term Infants

Growth charts (for the 3rd, 10th, 25th, 50th, 75th, 90th, and 97th percentiles, denoted as P3, P10, P25, P50, P75, P90, and P97, respectively) of length/height, weight, HC, and body mass index (BMI) of the preterm and term infants at 40–160 weeks PMA stratified by sex were constructed using Generalized Additive Models for Location, Scale, and Shape (GAMLSS) (15, 16) in R software (version 3.6.1). The selection of the GAMLSS model for each growth parameter of the preterm and term infants stratified by sex was based on the Akaike information criterion (AIC) (17) and the Bayesian information criterion (BIC) or Schwarz Bayesian criterion (SBC) (18). The final selected model for each growth parameter is shown in Supplementary Table 1.

### Comparisons of Growth Charts of the Preterm Infants, Term Infants, and WHO Standards

Difference values for P50 (the 50th percentile) of each growth parameter were calculated between the preterm and term infants, and between the preterm infants and WHO standards.

The Z-scores were assigned to 11 PMA clusters based on the PMA at the follow-up visit: PMA ≥ 40 and <44, ≥44 and <50, ≥50 and <56, ≥56 and <62, ≥62 and <68, ≥68 and <76, ≥76 and <84, ≥84 and <94, ≥94 and <112, ≥112 and <132, ≥132 and ≤ 160 weeks. The Z-scores were compared between the preterm and term infants, and we also observed whether the Z-scores were >0 (indicating higher growth levels than the WHO standards).

### Statistical Analysis

The statistical analysis was conducted using SPSS software version 21 (IBM Corp., Armonk, NY, USA). Continuous variables are presented as mean (SD), while categorical variables are presented as frequency (*n*) and percentage (%). Baseline characteristics were compared between the preterm and term infants, with independent-sample *t*-tests and chi-square tests being used to compare continuous and categorical variables, respectively. The level of significance was set at *P* ≤ 0.05.

## Results

### Baseline Characteristics

There were 5,459 sets of anthropometric measurements (each included length/height, weight, HC, and BMI) for 559 eligible preterm infants, and a further 15,185 for 1,596 term infants, from birth to 160 weeks PMA. That was, the mean of the follow-up visits was 9.77 for preterm infants and 9.51 for term infants (including that at birth). The comparison of the baseline characteristics between the preterm and term infants is shown in Table 1.

**Table 1:** Baseline characteristics of singleton preterm and term infants^Δ^^*^.

	**Preterm (*n* = 559)**	**Term (*n* = 1,596)**	***P***
PMA at birth	33.84 (2.93)	40.00 (0.00)	<0.001
Boys	326 (58.3%)	813 (50.9%)	0.002
Length at birth (cm)	45.46 (4.49)	50.70 (1.38)	<0.001
Weight at birth (kg)	2.35 (0.71)	3.54 (0.39)	<0.001
BMI at birth (kg/m^2^)	11.02 (2.00)	13.76 (1.21)	<0.001
HAZ at birth (INSR/S)^§^	0.59 (1.17)	0.69 (0.83)	0.059
WAZ at birth (INSR/S)^§^	0.38 (1.00)	0.49 (0.90)	0.014
First gestation	335 (59.9%)	1,382 (86.6%)	<0.001
First birth	404 (72.3%)	1,462 (91.6%)	<0.001
Cesarean section	301 (53.8%)	782 (44.6%)	<0.001
Maternal age (year)	31.20 (4.33)	29.30 (3.29)	<0.001
Paternal age (year)	32.66 (5.12)	30.97 (4.44)	<0.001
Maternal education: ≥college	490 (87.7%)	1,420 (89%)	0.221
Paternal education: ≥college	508 (90.9%)	1,443 (90.4%)	0.410
Maternal ethnicity: Han	554 (99.1%)	1,569 (98.3%)	0.125
Paternal ethnicity: Han	555 (99.3%)	1,574 (98.6%)	0.156
**PMA at birth subgroups**			
Extremely preterm ( ≤ 28 weeks)	47 (8.4%)	–	–
Moderate preterm (29–33 weeks)	115 (20.6%)	–	–
Late preterm (34–36 weeks)	397 (71.0%)	–	–
**Birth weight subgroups**			
ELBW (<1.0 kg)	28 (5.0%)^a^	0 (0%)	<0.001
VLBW (1.0–1.5 kg)	60 (10.7%)^a^	0 (0%)	
LBW (1.5–2.5 kg)	214 (38.3%)^a^	3 (0.2%)	
NBW (≥2.5 kg)	257 (46.0%)^a^	1593 (99.8%)	
**Intrauterine growth status subgroups (based on birth weight percentile)**^**§**^			
SGA (< P10)	29 (5.2%)^a^	44 (2.8%)	0.018
AGA (P10–P90)	438 (78.5%)	1,263 (79.1%)	
LGA (>P90)	91 (16.3%)^a^	289 (18.1%)	

Δ *AGA, appropriate for gestational age; ELBW, extremely low birth weight; HAZ, Z-score of length; INSR/S, INTERGROWTH-21st International Newborn Size References/Standards; LBW, low birth weight; LGA, large for gestational age; NBW, normal birth weight; PMA, postmenstrual age; SGA, small for gestational age; VLBW, very low birth weight; WAZ, Z-score of weight*.

* *Presented as mean (SD) or n (%). Independent-sample t-tests and chi-square tests were used to compare continuous and categorical variables between the preterm and term infants, respectively*.

a *Significant difference of subgroups (P < 0.05) according to the chi-square test*.

§ *Z-scores for preterm infants born at 23 weeks PMA could not be calculated according to the INSR/S (range of PMA: 24–42 weeks)*.

The mean PMA at birth for the preterm infants was 33.84 weeks (range, 23–36 weeks); 71.0% were late preterm infants, and almost half of them had normal birth weight (NBW). The general baseline characteristics of the preterm and term infants were similar. Almost all infants were of Han ethnicity (>98%), and most had well-educated parents (>85% had at least one parent with higher education). The proportions of boys and infants born by cesarean section were significantly higher among the preterm infants than the term infants. The parents of the preterm infants were older than those of the term infants, and there were fewer mothers experiencing their first gestation and first birth in the preterm infant cohort. The HAZ and WAZ at birth of both the preterm and term infants according to the INSR/S (14) were >0, which were lower in the preterm infants than the term infants, with statistically significant differences. Based on INSR/S birth weight percentiles, the proportion assessed as small for gestational age (SGA) was significantly higher among the preterm infants than the term infants, while the proportion assessed as large for gestational age (LGA) was significantly lower.

### Growth Charts for the Preterm and Term Infants

Growth charts (P3, P10, P25, P50, P75, P90, and P97) of length/height, weight, HC, and BMI for the preterm and term infants stratified by sex at 40–160 weeks PMA are shown in Supplementary Table 2 (preterm infants), Supplementary Table 3 (term infants), and Figures 2–9 (showing the P3, P50, and P97 growth curves).

**Figure 2:**
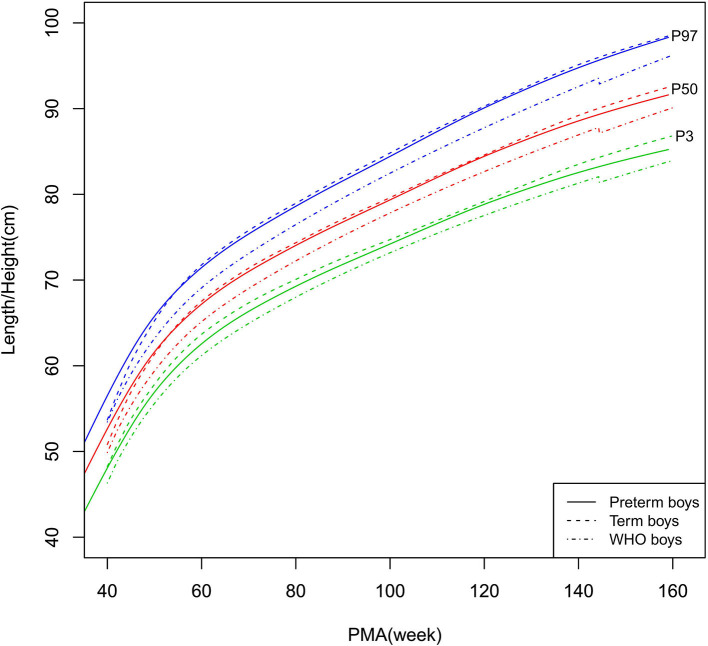
Length/height growth curves (P3, P50, and P97) of the preterm boys, term boys, and WHO standards.

**Figure 3:**
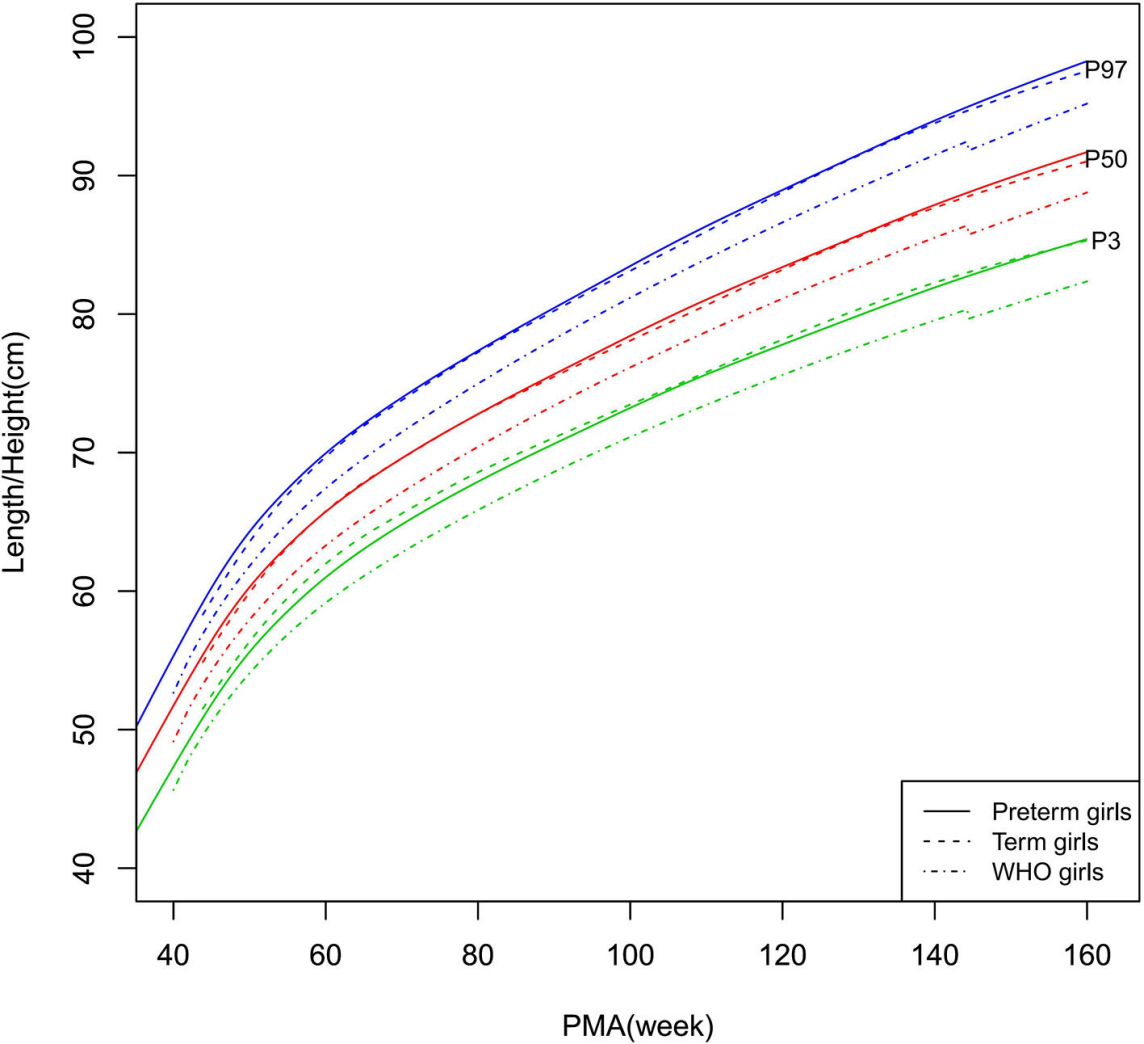
Length/height growth curves (P3, P50, and P97) of the preterm girls, term girls, and WHO standards.

**Figure 4:**
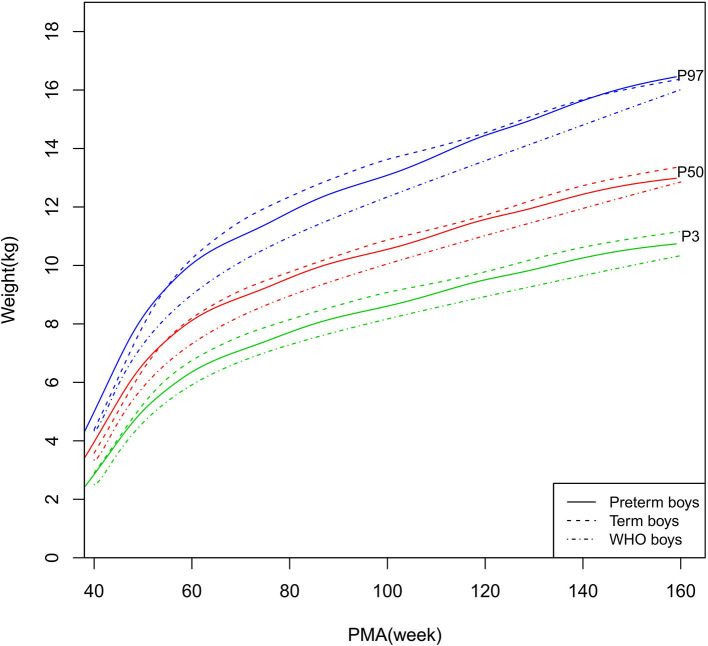
Weight growth curves (P3, P50, and P97) of the preterm boys, term boys, and WHO standards.

**Figure 5:**
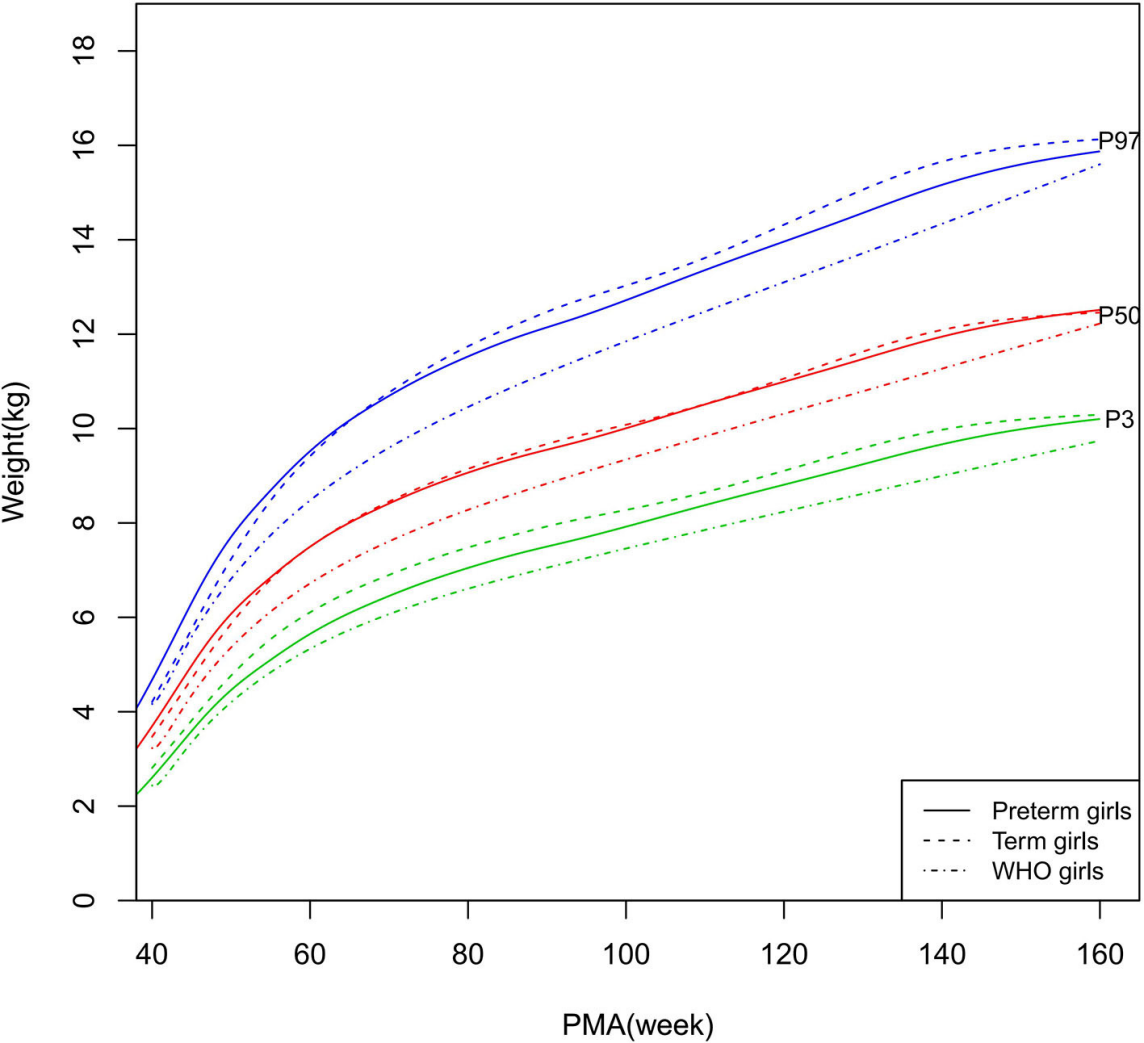
Weight growth curves (P3, P50, and P97) of the preterm girls, term girls, and WHO standards.

**Figure 6:**
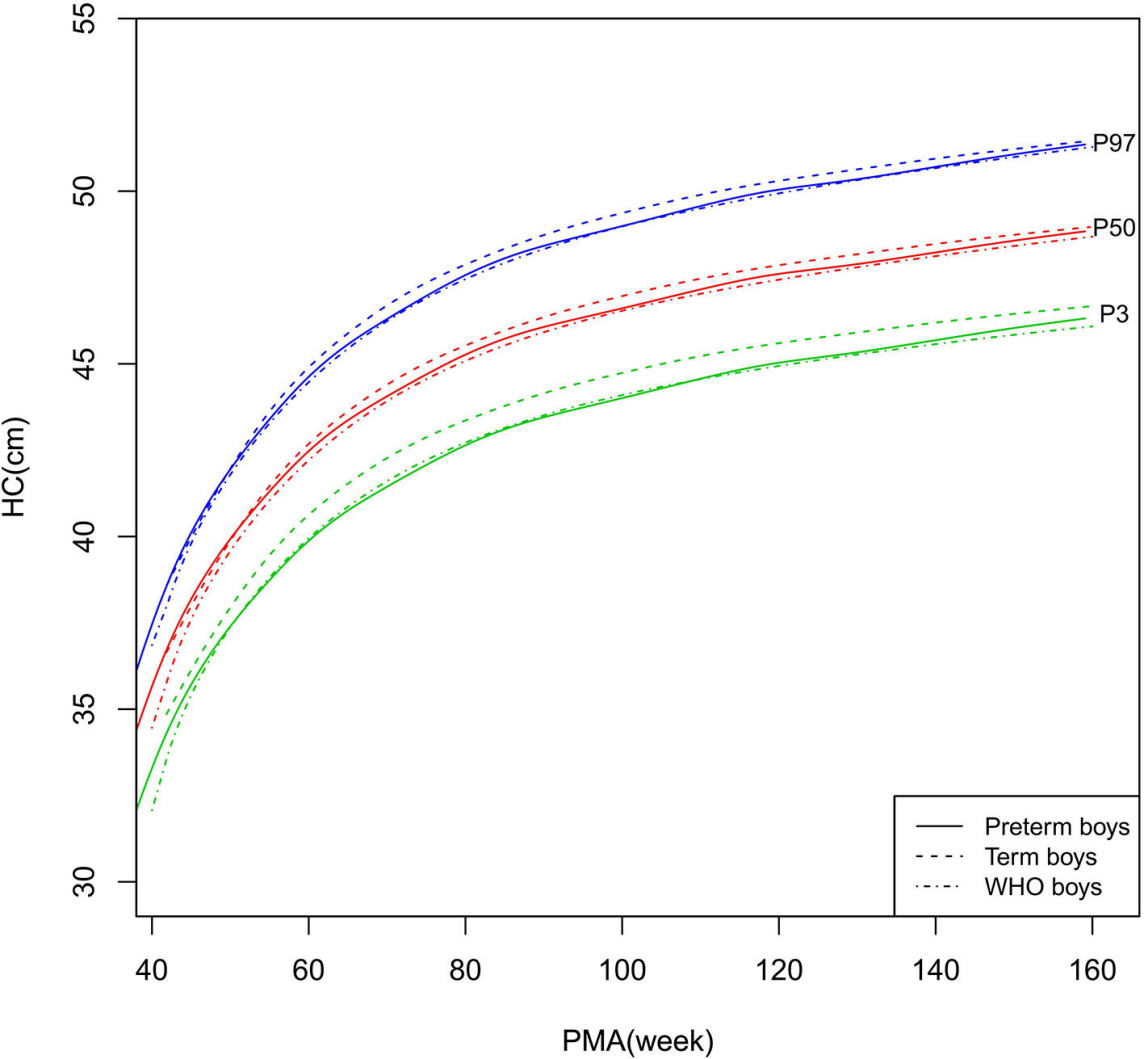
HC growth curves (P3, P50, and P97) of the preterm boys, term boys, and WHO standards. HC, head circumference.

**Figure 7:**
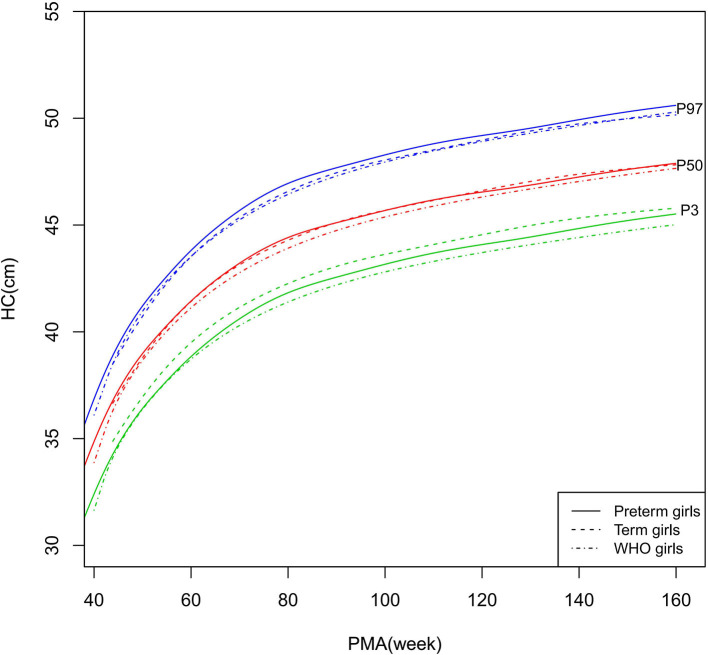
HC growth curves (P3, P50, and P97) of the preterm girls, term girls, and WHO standards. HC, head circumference.

**Figure 8:**
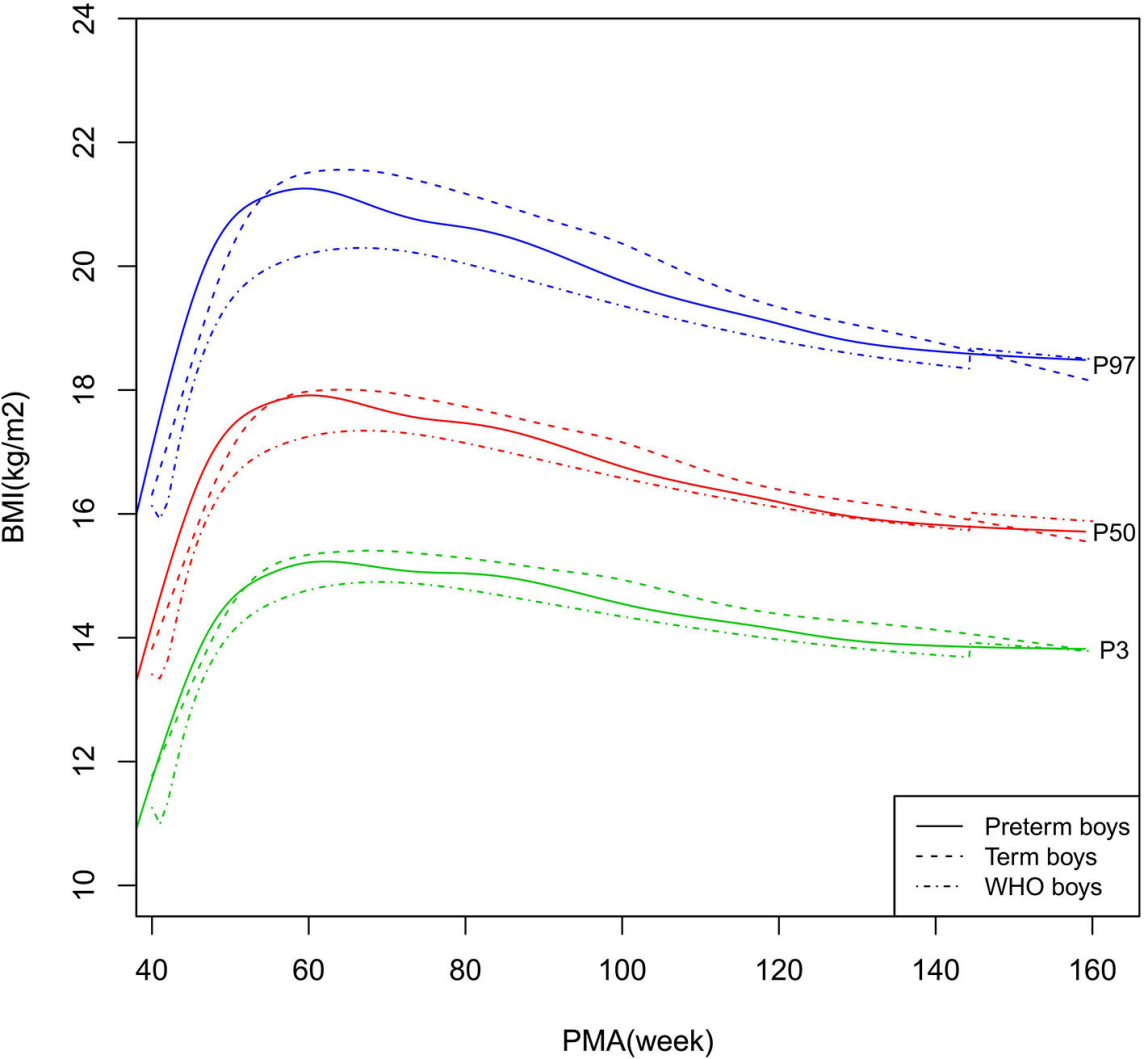
BMI growth curves (P3, P50, and P97) of the preterm boys, term boys, and WHO standards. BMI, Body Mass Index.

**Figure 9:**
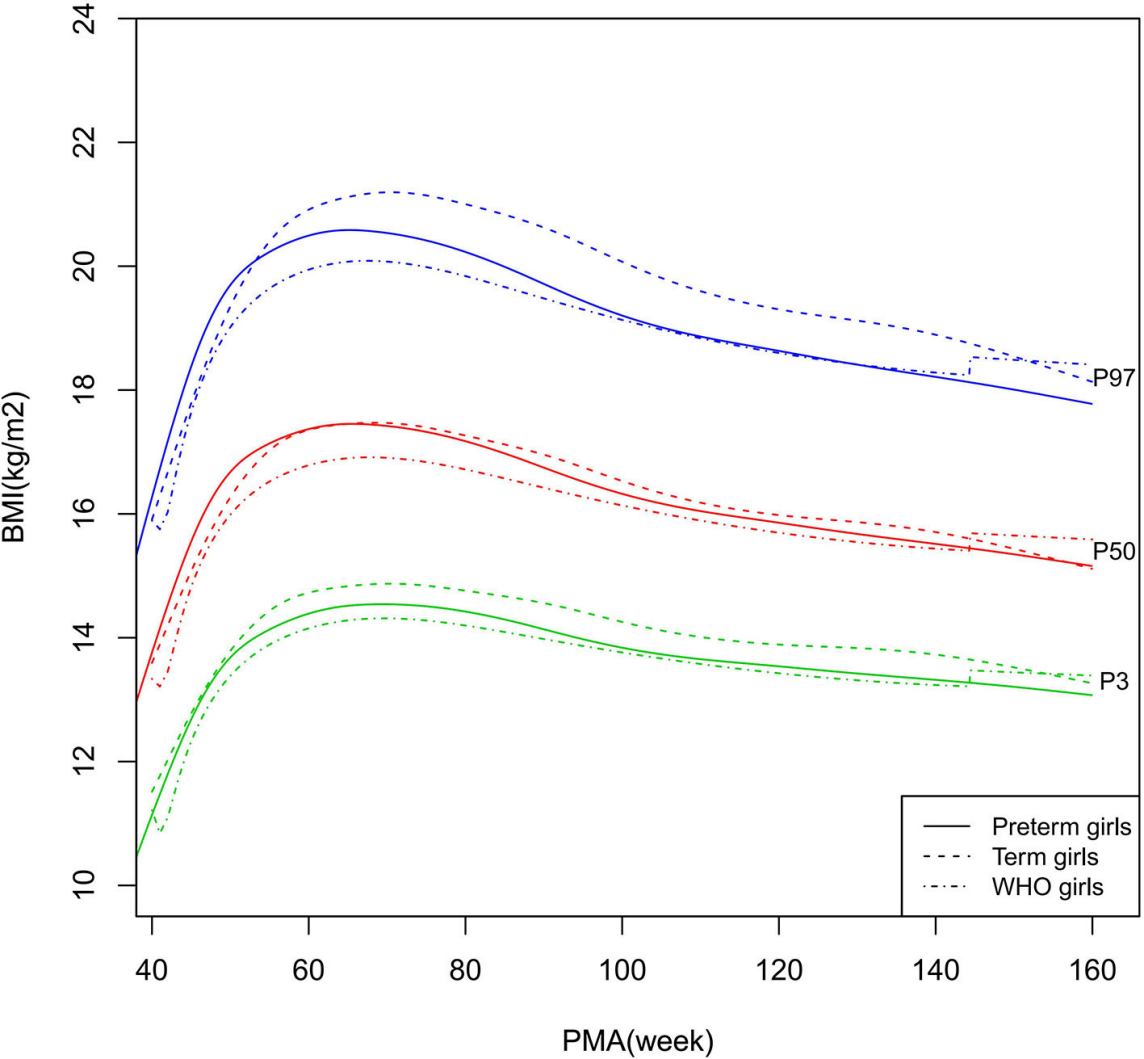
BMI growth curves (P3, P50, and P97) of the preterm girls, term girls, and WHO standards. BMI, Body Mass Index.

### Comparisons Among the Preterm Infants, Term Infants, and WHO Growth Standards

#### Differences Between the Preterm and Term Infants, and Between the Preterm Infants and WHO Standards

**Figure 10:**
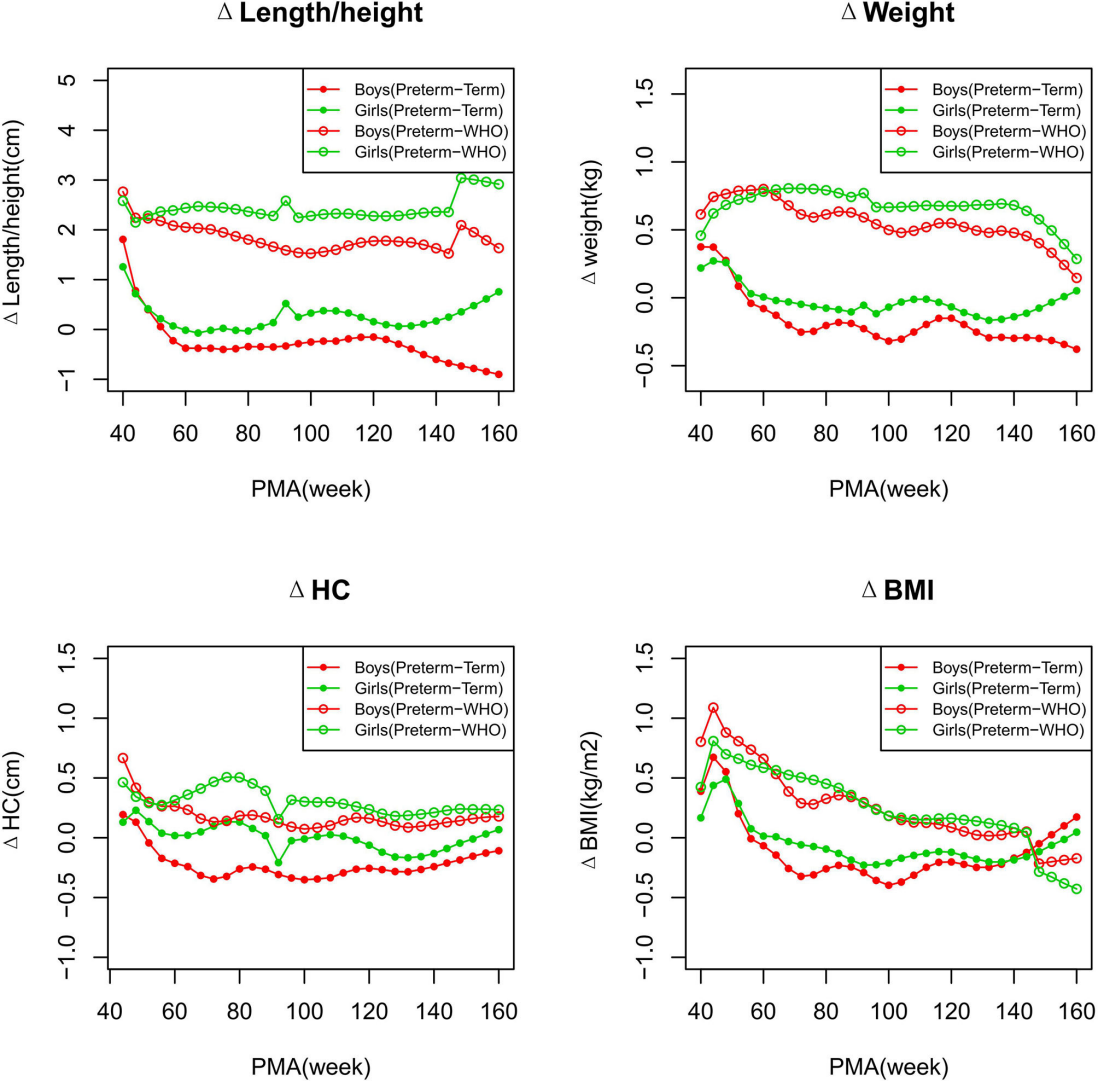
Differences between the preterm and term infants, and between the preterm infants and WHO standards. Differences are presented as Δlength/height, Δweight, ΔHC, and ΔBMI, which were calculated as the P50 values of the preterm infants minus those of the term infants or the WHO growth standards.

The difference values for P50 of each growth parameter between the preterm and term infants, and the preterm infants and WHO standards, are shown in Figure 10 and Supplementary Table 4.

##### Length/Height, Weight, and BMI

As shown in Figures 2–5, 8–10, compared to the term infants, the length/height, weight, and BMI of the preterm infants were larger at 40 weeks PMA. Thereafter, these parameters grew at a slower rate for preterm infants than term infants. At around 52–64 weeks PMA, the preterm infants were surpassed by the term infants. At 64–144 weeks PMA (equivalent to corrected 2 years old), the growth trajectories of the preterm infants were parallel to but slightly lower than those of the term infants except for the preterm girls' length/height, which was almost completely consistent with that of the term girls. The preterm girls had smaller differences than the preterm boys in length/height, weight, and BMI compared to their term counterparts. After 144 weeks PMA, the differences between the preterm and term infants tended to increase.

In contrast, at 40–144 weeks PMA, the preterm infants had a greater length, weight, and BMI than the WHO standards (Figures 2–5, 8–10 and Supplementary Table 4). The BMI of the preterm infants gradually approached the WHO standards and, at 100–144 weeks PMA, the difference was <0.20 kg/m^2^. After 144 weeks PMA, the weight of the preterm infants was rapidly approaching the WHO standards, the BMI became smaller than the WHO standards, while the height tended to even larger than the WHO standards.

##### HC

As shown in Figures 6–7, 10, regarding HC, both the preterm boys and girls had highly consistent growth trajectories with their term counterparts and the WHO standards at 40–160 weeks PMA. The preterm boys had smaller differences relative to the WHO standards, while the preterm girls had smaller differences relative to the term girls.

#### Z-Scores of the Preterm and Term Infants According to the WHO Standards

The Z-scores of each growth parameter for the preterm and term infants according to the WHO standards are shown in Table 2. The HAZ, WAZ, HCZ, and BAZ of the preterm and term infants at 40–160 weeks PMA were clearly >0, except for the BAZ of the term infants at 132–160 weeks PMA.

**Table 2:** Z-scores of the preterm and term infants according to the WHO standards^#^^Δ^.

**PMA cluster (weeks)**	***n***		**PMA (week)**		**HAZ**		**WAZ**		**HCZ**		**BAZ**	
	**Preterm**	**Term**	**Preterm**	**Term**	**Preterm**	**Term**	**Preterm**	**Term**	**Preterm**	**Term**	**Preterm**	**Term**
≥40 and <44	429	1598	41.39 (1.12)^¶^	40.00 (0.11)	1.20 (1.19)^¶^	0.63 (0.74)	1.13 (1.11)^¶^	0.49 (0.78)	0.73 (1.00)	–	0.74 (1.10)^¶^	0.25 (0.92)
≥44 and <50	408	1297	47.83 (1.63)^¶^	45.72 (1.05)	1.16 (1.20)^¶^	0.85 (0.95)	1.08 (1.06)^¶^	0.60 (0.80)	0.29 (1.01)	0.25 (0.84)	0.64 (1.00)^¶^	0.20 (0.91)
≥50 and <56	490	1340	52.97 (1.57)^¶^	53.55 (0.85)	0.99 (1.15)	1.08 (0.94)	0.84 (1.14)	0.91 (0.92)	0.13 (1.00)^*^	0.25 (0.88)	0.39 (1.07)	0.42 (1.05)
≥56 and <62	461	1371	59.02 (1.79)	58.87 (1.69)	1.04 (1.11)	1.15 (0.96)	0.90 (1.14)	1.00 (0.99)	0.22 (1.07)	0.32 (0.87)	0.43 (1.09)	0.47 (1.06)
≥62 and <68	375	1770	64.17 (1.78)^¶^	65.30 (1.82)	0.97 (1.06)^*^	1.13 (0.98)	0.80 (1.07)^§^	0.96 (1.00)	0.17 (1.00)^§^	0.35 (0.90)	0.34 (1.02)	0.43 (1.05)
≥68 and <76	447	1224	71.87 (1.99)^¶^	73.18 (2.70)	0.95 (1.07)^*^	1.07 (0.98)	0.71 (1.04)^¶^	0.93 (1.00)	0.20 (1.02)^§^	0.36 (0.91)	0.24 (1.02)^¶^	0.44 (1.04)
≥76 and <84	342	1062	79.78 (1.94)^§^	80.15 (2.94)	0.85 (1.10)^*^	0.99 (0.99)	0.66 (1.06)^¶^	0.88 (1.02)	0.23 (1.01)	0.33 (0.92)	0.25 (1.02)^§^	0.44 (1.05)
≥84 and <94	481	1601	88.79 (2.45)^¶^	90.05 (3.54)	0.78 (1.07)	0.87 (1.01)	0.64 (1.03)^*^	0.78 (0.97)	0.17 (1.02)^§^	0.33 (0.90)	0.29 (1.03)^*^	0.40 (1.03)
≥94 and <112	418	1297	102.64 (4.59)	102.84 (5.22)	0.71 (1.08)	0.73 (1.05)	0.45 (0.98)^¶^	0.70 (0.98)	0.08 (1.03)^¶^	0.30 (0.92)	0.07 (0.99)^¶^	0.40 (1.02)
≥112 and <132	454	1402	117.49 (4.89)^¶^	119.88 (3.08)	0.71 (1.07)	0.75 (1.02)	0.51 (0.98)	0.60 (0.93)	0.17 (0.99)^*^	0.29 (0.90)	0.14 (0.99)^*^	0.25 (0.98)
≥132 and ≤ 160	520	1223	142.11 (4.85)^¶^	146.01 (4.26)	0.67 (1.06)^¶^	0.87 (1.00)	0.42 (0.99)^*^	0.53 (0.93)	0.11 (0.98)^§^	0.25 (0.87)	0.03 (1.00)	−0.01 (0.99)

* P <0.05,

§ P <0.01,

¶ *P <0.001*.

# *BAZ, Z-score of BMI; HAZ, Z-score of length/height; HCZ, Z-score of head circumference; PMA, postmenstrual age; WAZ, Z-score of weight*.

Δ *Z-scores of the preterm infants were calculated after correction for prematurity. HCZ values of the term infants at 40–44 weeks PMA were absent, as HC was not measured at birth in this study*.

##### Z-Scores of the Preterm Infants

For preterm infants, the largest mean values of HAZ, WAZ, HCZ, and BAZ were 1.20, 1.13, 0.73, and 0.74, respectively, at 40–44 weeks PMA. HAZ, WAZ, and BAZ then gradually decreased. At 132–160 weeks PMA, HAZ, WAZ, and BAZ were at their lowest values (0.67, 0.42, and 0.03, respectively). HCZ rapidly decreased from 0.73 at 40–44 weeks PMA to 0.29 at 44–50 weeks PMA, and it then fluctuated in the range of 0.08–0.23 at 50–160 weeks PMA.

##### Comparison of Z-Scores Between the Preterm and Term Infants

Although there was a difference in the mean PMA between the preterm and term infants in each PMA cluster, we still compared the Z-scores of the preterm and term infants as, unlike the linear relationship between growth parameters and PMA, there is no such direct linear relationship between the Z-scores of growth parameters and PMA.

##### HAZ, WAZ, and BAZ

Before 50 weeks PMA, the HAZ, WAZ, and BAZ were all significantly larger for the preterm infants than the term infants. At 50–62 weeks PMA, the HAZ, WAZ, and BAZ were similar between the preterm and term infants, but thereafter, the preterm infants mostly had smaller values (except for BAZ at 132–160 weeks PMA) than the term infants (statistical significance existed for HAZ at 62–84 and 132–160 weeks PMA, WAZ at 62–112 and 132–160 weeks PMA, and BAZ at 68–132 weeks PMA).

##### HCZ

At 44–50 weeks PMA, the HCZ of the preterm infants was similar to that of the term infants. Subsequently, the term infants had larger HCZ than the preterm infants (statistical significance existed at 50–56, 62–76, and 84–160 weeks PMA).

## Discussion

Our retrospective cohort study benefits from the stringent inclusion and exclusion criteria used for the preterm infants and their control term infants. For example, all subjects were singletons born during the same period of time at the same center and had a sufficient follow-up frequency and duration. Furthermore, the selection of control subjects who were born at strictly 40 weeks PMA took into account the fact that term infants born at 37–41 weeks PMA might have varying growth trajectories. Thus, the growth level of the selected term infants represented the ideal growth level of healthy local children. In addition, we selected PMA as the age variable, which allowed for accurate and specific comparisons among the preterm infants, term infants, and the WHO standards.

The study demonstrated that the postnatal growth trajectories of the preterm infants were different from the WHO standards and similar but not identical to those of their term counterparts. Overall, the length/height, weight, HC, and BMI of the preterm infants were larger than the WHO standards, especially length/height and weight, of which the gap between the preterm infants and the WHO standards during the corrected term to 2 years old could span two adjacent centile curves. The growth trajectories of the preterm infants were more consistent with their term counterparts than the WHO standards, especially for length/height and weight after around 50 weeks PMA.

Kang et al. lately published a multi-center cohort study conducted in Sichuan, China, which also made a comparison of the postnatal growth between preterm and term infants using longitudinal growth data during 40–88 weeks PMA (19). Different from our study, twins and infants with only one follow-up visit were all included in this study. However, the results obtained in this study were in good agreement with ours: compared with international growth standards, preterm infants had higher growth levels than the INTERGROWTH-21st International Preterm Postnatal Growth Standards (IPPGS) and WHO standards; and compared with term counterparts, preterm infants had higher growth levels at corrected term, were caught-up by term infants at around 1–3 corrected month age, and had consistent growth with term counterparts afterward (19). In addition, our previous study of late preterm infants also demonstrated increased growth compared to the widely-used Fenton reference during birth and corrected term age (13).

The conclusion that preterm infants had higher postnatal growth than international growth charts and consistent growth with term counterparts, was different from most previous studies, which concluded that children born preterm tended to exhibit less growth than children born full-term and that these children had an increased risk of growth retardation (20–22). These discrepancies may be in part attributable to the differences in methods. Most previous studies concentrated on very preterm infants and/or very low birth weight (VLBW)/extremely low birth weight (ELBW) infants (20–22). While our study and Kang et al.'s study concentrated on contemporary Chinese preterm cohorts involving a more complete range of gestational age for preterm infants (19). The different growth trajectories between preterm and term infants during early postnatal life (corrected term and 1–3 months) might be due to the growth fluctuations of the term infants around term, including the physiological weight loss after birth and the growth deceleration of the fetus just prior to 40 weeks, which was not experienced by preterm infants during this period (23).

These findings indicated that Chinese preterm infants might also have higher growth levels than international growth standards during the first 2 years of life, and these findings include some that were found in previous population-based studies with term infants in China. For example, both the Chinese National Growth Survey (CNGS) 2005 and 2015, which constructed the growth charts of Chinese children under 7 years of age with cross-sectional data of urban singleton term-born children from nine cities in China, had concluded the higher growth levels than the WHO standards (24, 25). The HAZ and WAZ of term infants in CNGS 2015 were 0.47 (1.01) and 0.48 (0.93), respectively, before 1 year of age; and 0.38 (1.02) and 0.35 (0.91), respectively, at 1–2 years of age (24). Our singleton term infants demonstrated higher levels than WHO standards and consistent growth trajectories to CNGS 2015 during the first 2 years of life but slightly higher than the latter. Furthermore, term infants in Kang et al.'s study also showed higher length and weight growth levels than the WHO standards during the first year of life (19).

Given the concern of the relationship between excessive catch-up growth and subsequent development of metabolic problems later in life (1), this higher postnatal growth level of our preterm infants than what has been shown in previous studies and international growth charts warrants further analysis of etiology. Our study provided some evidence that the higher growth level regarding the length/height and weight of the preterm infants relative to the WHO standards might be a manifestation of the children reaching their local population-specific growth potential rather than excessive growth. First, the growth trajectories of the length/height and weight of our preterm infants were rather close to those of the term infants at the same center after around 50 weeks PMA. Second, both preterm and term infants had higher growth levels than the WHO standards. Third, the BMI (an indicator of body composition) of the preterm infants gradually approached that of the term infants and the WHO standards, and HC (an indicator of later neurodevelopment) of the preterm infants was quite consistent with that of the term counterparts and the WHO standards. In addition, the similarities between the findings of Kang et al.'s study (19) and CNGS (24, 25) with our study in the comparison of preterm and term infants using international growth standards further indicated the possible physiological origin of this higher growth level of our preterm infants.

As we have known, there is a trend of constructing and using international references/standards of optimal physiological growth in the evaluation of growth level and health status of children worldwide over the past decades, the most prominent of which is the WHO standards for term infants (26, 27), Fenton reference (28) and IPPGS for preterm infants (7). The CNGS was also constructed as a pooled reference from nine cities in northern, central, and southern China (24, 25). It was based on a controversial assumption that there would be no differences internationally or regionally among countries, regions, or racial/ethnic groups in growth when conditions were optimal (29–31). However, there remain concerns that human growth may be strongly genetically determined, which results in significant variations in stature, HC, and body compositions among different populations (10, 13). For example, the heritability of height is estimated to be around 80% (10). In fact, in the WHO Multicentre Growth Reference Study (MGRS) (27) and the INTERGROWTH-21st study (7, 29, 32), the differences among countries/regions were interpreted as small enough to not be meaningful (and formal hypothesis testing was not conducted), so the final decision was to create a single growth standard. However, the Eunice Kennedy Shriver National Institute of Child Health and Human Development Study demonstrated differences in the fetal growth of different racial/ethnic groups (29, 33). In China, the CNGS 2005 and 2015 both demonstrated obvious regional differences, with obviously higher growth levels in the northern and central regions relative to the southern region (24, 25). The IPPGS was also based on pooled longitudinal data of eight geographically defined populations, of which the sample size (201 singleton preterm infants) might not be large enough for reliable conclusions as to whether there were differences among different populations (7). The current consensus is that a pooled standard/reference should only be derived if there are no racial/ethnic/regional differences. Otherwise, population-based growth references/standards based on children with the same genetic and environmental background should be used in addition to the international growth charts (10, 29–31). Specifically in our study, most preterm infants and their term counterparts were born and live in Jinan, Shandong Province, which has always been an area in China where people have tended to be tall. Thus, the increased growth level of the preterm and term infants compared to the international growth charts in this study might indicate achievement of the local population-specific growth potential rather than excessive growth.

There are some limitations to this study. First, our study was conducted in a single center in Jinan, China, although the First Affiliated Hospital of Shandong First Medical University is a tertiary public hospital that covers the prevention and treatment of maternal and child diseases in Shandong province, China. Second, given that the mean gestational age among the preterm infants was 33.84 weeks and the fact that 71% of the preterm infants were late preterm infants, our study might not be sufficiently powered to evaluate the specific growth of extremely preterm, moderate preterm, and ELBW/VLBW infants. Third, infants aged <4 months in the WHO cohort were exclusively breastfed (34), while it was only recommended that our preterm infants should be exclusively breastfed before a corrected age of 4–6 months, and the proportion of infants that were exclusively breastfed was not documented. This means that we cannot firmly conclude that the growth of our cohort was intrinsically or

genetically different than that of the WHO cohort, which was thought to represent the ideal growth pattern.

In a future study, we will establish a birth cohort of preterm infants with a larger sample size, more detailed documentation of growth data and potential bias factors, and increased duration of follow-up, and we will construct stable and reliable growth charts stratified by sex and PMA at birth.

In conclusion, the singleton preterm infants in a single-center in China had specific growth trajectories that were obviously higher than the WHO standards and similar but not identical to their term counterparts during the first 2 years of life, which reemphasizes the necessity of constructing growth charts especially for a local Chinese population of singleton preterm infants.

## Data Availability Statement

The original contributions presented in the study are included in the article/Supplementary Material, further inquiries can be directed to the corresponding author/s.

## Ethics Statement

The studies involving human participants were reviewed and approved by Medical Ethics Committee of the First Affiliated Hospital of Shandong First Medical University. Written informed consent to participate in this study was provided by the participants' legal guardian/next of kin.

## Author Contributions

LZ made a major contribution to the conception and design of the study, the collection, analysis, and interpretation of the data, and the writing and revision of the manuscript. J-GL made a substantial contribution to the data analysis. SL revised the manuscript for important intellectual content. JS, N-NG, QW, H-YZ, H-JL, X-DC, and YC contributed to the acquisition of data. YL made a substantial contribution to the supervision and project administration of the study and the writing and revision of the manuscript. All authors read and approved the final manuscript.

## Conflict of Interest

The authors declare that the research was conducted in the absence of any commercial or financial relationships that could be construed as a potential conflict of interest.
