# Supplementary material for: Comparison of Postnatal Growth Charts of Singleton Preterm and Term Infants Using World Health Organization Standards at 40–160 Weeks Postmenstrual Age: A Chinese Single-Center Retrospective Cohort Study
Source: Front Pediatr. 2021 Mar 15;9:595882. doi: 10.3389/fped.2021.595882 (PMC8005644; doi:10.3389/fped.2021.595882)
Supplement: Supplementary file 1 [file Data_Sheet_1.ZIP › Supplementary tables/Supplementary Table 1 GAMLSS model of preterm and term infants.docx]

Table S1 GAMLSS models of preterm and term infants stratified by sex^△*^.

| Growth Parameters | Preterm Infants | | Term Infants | |
| --- | --- | --- | --- | --- |
|  | Boys | Girls | Boys | Girls |
| Length/Height | BCCGo | BCCGo | BCTo | BCTo |
| Weight | BCPEo | BCPEo | BCTo | BCPEo |
| HC | BCCGo | BCCGo | BCTo | BCTo |
| BMI | BCCGo | BCCGo | BCTo | BCTo |

△Abbreviation: BCCGo, Box-Cox Cole-Green orig. ; BCPEo: Box-Cox power exponential distribution orig. ; BCTo: Box-Cox t orig. ; GAMLSS: Generalized Additive Models for Location, Scale and Shape; HC: Head circumference.

* Model selection was according to the Akaike information criterion (AIC) and the Bayesian information criterion (BIC) or Schwarz Bayesian criterion (SBC).
