# Supplementary material for: Comparison of Postnatal Growth Charts of Singleton Preterm and Term Infants Using World Health Organization Standards at 40–160 Weeks Postmenstrual Age: A Chinese Single-Center Retrospective Cohort Study
Source: Front Pediatr. 2021 Mar 15;9:595882. doi: 10.3389/fped.2021.595882 (PMC8005644; doi:10.3389/fped.2021.595882)
Supplement: Supplementary file 1 [file Data_Sheet_1.ZIP › Supplementary tables/Supplementary Table 4 Difference values between preterm and term, preterm and WHO.docx]

Table S4 Difference values between preterm and term infants, preterm infants and WHO standards*.

| PMA (week) | ΔLength/height (Preterm-Term, cm) | | ΔLength/height (Preterm-WHO, cm) | | ΔWeight (Preterm-Term, kg) | | ΔWeight (Preterm-WHO, kg) | | ΔHC  (Preterm-Term, cm) | | ΔHC  (Preterm-WHO, cm) | | ΔBMI  (Preterm-Term, kg/m^2^) | | ΔBMI  (Preterm-WHO, kg/m^2^) | |
| --- | --- | --- | --- | --- | --- | --- | --- | --- | --- | --- | --- | --- | --- | --- | --- | --- |
|  | Boys | Girls | Boys | Girls | Boys | Girls | Boys | Girls | Boys | Girls | Boys | Girls | Boys | Girls | Boys | Girls |
| 40 | 1.81 | 1.26 | 2.77 | 2.58 | 0.37 | 0.22 | 0.61 | 0.46 | - | - | 1.20 | 0.98 | 0.39 | 0.17 | 0.80 | 0.42 |
| 44 | 0.78 | 0.72 | 2.24 | 2.15 | 0.37 | 0.27 | 0.74 | 0.62 | 0.19 | 0.13 | 0.67 | 0.46 | 0.67 | 0.44 | 1.09 | 0.81 |
| 48 | 0.40 | 0.42 | 2.23 | 2.28 | 0.28 | 0.26 | 0.77 | 0.68 | 0.13 | 0.23 | 0.42 | 0.34 | 0.55 | 0.49 | 0.88 | 0.70 |
| 52 | 0.06 | 0.22 | 2.18 | 2.37 | 0.09 | 0.14 | 0.79 | 0.72 | -0.04 | 0.14 | 0.30 | 0.29 | 0.20 | 0.29 | 0.81 | 0.66 |
| 56 | -0.23 | 0.07 | 2.09 | 2.39 | -0.04 | 0.03 | 0.79 | 0.74 | -0.17 | 0.04 | 0.26 | 0.27 | -0.01 | 0.08 | 0.74 | 0.61 |
| 60 | -0.38 | -0.01 | 2.05 | 2.44 | -0.08 | 0.01 | 0.80 | 0.78 | -0.21 | 0.02 | 0.26 | 0.31 | -0.07 | 0.01 | 0.66 | 0.59 |
| 64 | -0.38 | -0.07 | 2.04 | 2.47 | -0.13 | -0.02 | 0.75 | 0.80 | -0.24 | 0.02 | 0.23 | 0.36 | -0.14 | 0.01 | 0.53 | 0.56 |
| 68 | -0.38 | -0.02 | 2.01 | 2.46 | -0.20 | -0.03 | 0.68 | 0.81 | -0.32 | 0.05 | 0.16 | 0.41 | -0.26 | -0.03 | 0.39 | 0.53 |
| 72 | -0.40 | 0.02 | 1.95 | 2.45 | -0.25 | -0.05 | 0.61 | 0.80 | -0.35 | 0.10 | 0.13 | 0.47 | -0.32 | -0.06 | 0.29 | 0.50 |
| 76 | -0.39 | -0.02 | 1.87 | 2.42 | -0.25 | -0.06 | 0.59 | 0.80 | -0.32 | 0.13 | 0.14 | 0.51 | -0.31 | -0.07 | 0.28 | 0.48 |
| 80 | -0.34 | -0.03 | 1.81 | 2.37 | -0.20 | -0.08 | 0.61 | 0.79 | -0.26 | 0.13 | 0.19 | 0.51 | -0.26 | -0.09 | 0.33 | 0.45 |
| 84 | -0.35 | 0.06 | 1.74 | 2.32 | -0.18 | -0.09 | 0.64 | 0.77 | -0.24 | 0.08 | 0.19 | 0.45 | -0.23 | -0.13 | 0.36 | 0.42 |
| 88 | -0.35 | 0.14 | 1.66 | 2.28 | -0.19 | -0.10 | 0.63 | 0.74 | -0.26 | 0.02 | 0.17 | 0.39 | -0.24 | -0.19 | 0.34 | 0.36 |
| 92 | -0.33 | 0.52 | 1.59 | 2.58 | -0.23 | -0.05 | 0.59 | 0.77 | -0.31 | -0.21 | 0.13 | 0.16 | -0.29 | -0.23 | 0.30 | 0.29 |
| 96 | -0.28 | 0.25 | 1.54 | 2.25 | -0.28 | -0.12 | 0.54 | 0.67 | -0.34 | -0.02 | 0.09 | 0.32 | -0.36 | -0.23 | 0.24 | 0.23 |
| 100 | -0.25 | 0.33 | 1.52 | 2.28 | -0.32 | -0.07 | 0.50 | 0.67 | -0.35 | -0.01 | 0.07 | 0.30 | -0.40 | -0.21 | 0.18 | 0.18 |
| 104 | -0.23 | 0.38 | 1.56 | 2.31 | -0.30 | -0.03 | 0.48 | 0.67 | -0.35 | 0.01 | 0.08 | 0.30 | -0.37 | -0.17 | 0.15 | 0.17 |
| 108 | -0.23 | 0.37 | 1.60 | 2.33 | -0.25 | -0.01 | 0.49 | 0.67 | -0.33 | 0.03 | 0.10 | 0.30 | -0.31 | -0.15 | 0.13 | 0.15 |
| 112 | -0.19 | 0.33 | 1.69 | 2.33 | -0.20 | -0.01 | 0.52 | 0.68 | -0.29 | 0.01 | 0.14 | 0.28 | -0.25 | -0.13 | 0.12 | 0.15 |
| 116 | -0.16 | 0.24 | 1.75 | 2.30 | -0.15 | -0.03 | 0.55 | 0.68 | -0.26 | -0.02 | 0.17 | 0.26 | -0.21 | -0.12 | 0.11 | 0.16 |
| 120 | -0.15 | 0.16 | 1.78 | 2.28 | -0.15 | -0.07 | 0.55 | 0.68 | -0.26 | -0.06 | 0.16 | 0.24 | -0.20 | -0.12 | 0.09 | 0.16 |
| 124 | -0.20 | 0.10 | 1.78 | 2.28 | -0.20 | -0.11 | 0.52 | 0.67 | -0.27 | -0.12 | 0.13 | 0.20 | -0.22 | -0.15 | 0.05 | 0.15 |
| 128 | -0.29 | 0.06 | 1.77 | 2.29 | -0.25 | -0.14 | 0.50 | 0.68 | -0.28 | -0.16 | 0.10 | 0.18 | -0.25 | -0.18 | 0.02 | 0.14 |
| 132 | -0.39 | 0.07 | 1.75 | 2.32 | -0.29 | -0.17 | 0.48 | 0.68 | -0.29 | -0.17 | 0.09 | 0.19 | -0.25 | -0.20 | 0.02 | 0.12 |
| 136 | -0.50 | 0.11 | 1.70 | 2.34 | -0.29 | -0.16 | 0.49 | 0.69 | -0.27 | -0.16 | 0.10 | 0.20 | -0.22 | -0.20 | 0.02 | 0.11 |
| 140 | -0.60 | 0.17 | 1.63 | 2.36 | -0.30 | -0.14 | 0.48 | 0.68 | -0.24 | -0.13 | 0.11 | 0.21 | -0.17 | -0.19 | 0.04 | 0.08 |
| 144 | -0.68 | 0.25 | 1.52 | 2.36 | -0.29 | -0.11 | 0.46 | 0.64 | -0.21 | -0.09 | 0.13 | 0.23 | -0.12 | -0.16 | 0.05 | 0.04 |
| 148 | -0.73 | 0.35 | 2.10 | 3.04 | -0.30 | -0.08 | 0.40 | 0.58 | -0.19 | -0.05 | 0.14 | 0.24 | -0.05 | -0.12 | -0.22 | -0.28 |
| 152 | -0.78 | 0.48 | 1.95 | 3.01 | -0.31 | -0.03 | 0.33 | 0.50 | -0.15 | -0.01 | 0.16 | 0.24 | 0.02 | -0.06 | -0.20 | -0.33 |
| 156 | -0.85 | 0.61 | 1.79 | 2.97 | -0.34 | 0.01 | 0.24 | 0.40 | -0.13 | 0.03 | 0.17 | 0.24 | 0.10 | -0.01 | -0.19 | -0.38 |
| 160 | -0.90 | 0.76 | 1.63 | 2.92 | -0.38 | 0.05 | 0.15 | 0.29 | -0.11 | 0.07 | 0.18 | 0.23 | 0.17 | 0.05 | -0.17 | -0.43 |

*Difference values were demonstrated as ΔLength/height, ΔWeight, ΔHC andΔBMI, which were calculated as the P50 values of preterm infants minus those of term infants and WHO growth standards.
